# Re-defining the human embryo: A legal perspective on the creation of embryos in research

**DOI:** 10.1038/s44319-023-00034-0

**Published:** 2024-01-02

**Authors:** Iñigo De Miguel Beriain, Jon Rueda, Adrian Villalba

**Affiliations:** 1 UPV/EHU, Leioa, Spain; 2 Basque Foundation of Science, Bilbao, Spain; 3 University of California-San Diego, Institute for Practical Ethics, San Diego, California 92109 USA; 4 University of Granada, Granada, Spain; 5 Université Paris Cité, Paris, France

**Keywords:** Development, Economics, Law & Politics

## Abstract

A universal legal definition of an embryo as a basis for regulation would create legal certainty for scientists and benefit international research cooperation.

**Figure:**
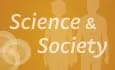

The notion of the human embryo is not immutable. Various scientific and technological breakthroughs in reproductive biology have compelled us to revisit the definition of the human embryo during the past 2 decades. Somatic cell nuclear transfer, oocyte haploidisation and, more recently, human stem cell-derived embryo models have challenged this scientific term, which has both ethical and legal repercussions. Here, we offer a legal perspective to identify a universally accepted definition of ‘embryo’ which could help to ease and unify the regulation of such entities in different countries.

## Historical perspective

Scientific advances in reproductive biology have made impressive achievements that seemed impossible thirty years ago (Villalba et al, 2023). The creation of embryos through techniques such as somatic cell nuclear transfer (SCNT) in the 1990s was only the first step in a great chain of milestones. SCNT challenged conventional notions of development, demonstrating the possibility of embryogenesis without fertilization involving two opposite-sex gametes. Nuclear transfer methods have since demonstrated their efficacy across a wide range of mammalian species, including non-human primates, prompting speculation about their applicability to human cells.

SCNT challenged conventional notions of development, demonstrating the possibility of embryogenesis without fertilization involving two opposite-sex gametes.

Lately, other technologies have emerged, as exemplified by the successful generation of mice through the merging of two oocytes in the absence of sperm. To do that, researchers combined haploidization to reduce the genetic dosage of each oocyte with genetic manipulation of one of the eggs to mimic male genetic imprinting. More recently, various groups have developed human embryo models from pluripotent stem cells to replicate early human embryonic structures in vitro (Villalba et al, 2023). These models themselves contain different pluripotent stem cell lines, leading to both embryonic and extraembryonic cell fates and recapitulating key histological features of preimplantation embryos.

… the precise delineation of what a human embryo is and its distinction from other, similar entities has assumed paramount significance.

We are, therefore, at a moment in which biotechnology can make a decisive contribution to improving our knowledge and control of the early stages of human life. However, this scenario generates challenging controversies from an ethical and legal point of view. This is not surprising at all—few biotechnological topics are more controversial than those involving human embryos. However, there is one aspect that remains unexplored in recent debates. Even though the moral status of the embryo has been a crucial issue in public discussions, the precise delineation of what a human embryo is and its distinction from other, similar entities has assumed paramount significance. This is not a merely a semantic problem, but a normative one, which has important practical consequences for research.

As a recent comment in *Nature* on an article published in *Cell* by Rivron et al (Rivron et al, 2023) stated, “it is time for a redefinition of the human embryo” (Ball, 2023). The problem is that the definition proposed by these authors—“a group of human cells supported by elements fulfilling extraembryonic and uterine functions that, combined, have the potential to form a fetus” (Rivron et al, 2023, p. 3548)—is not a sound definition, at least from the point of view of the law.

A universal and generally accepted legal definition of an embryo around a more rational interpretations would therefore help to alleviate legal uncertainties and harmonize regulations.

In this brief commentary, we will explain why this is not an optimal legal definition of an embryo and propose an alternative. This is a matter of particular importance because the different legal definitions of an embryo in different countries is problematic for research in particular for international collaborations among research groups. Legally, an embryo is not the same in Germany—where only the fertilized ovum is considered as such after the fertilization process has been completed—as it is in Spain—where a distinction is being made between embryo and pre-embryo—or in the Netherlands where it is a cell or group of cells with the potential to develop into an embryo. These diverse definitions not only represent a failure in axiological terms but also generate serious difficulties for research as the legal concept of the embryo informs national regulations on research with human embryos or embryonic stem cells. It does not only affect international research teams but also researchers who move from one country to another where they may no longer be permitted to continue their work. A universal and generally accepted legal definition of an embryo around a more rational interpretations would therefore help to alleviate legal uncertainties and harmonize regulations.

## Two competing definitions of human embryo

Broadly speaking, there are two main perspectives that inform the definition of an embryo. The first and older one considers the embryo as the result of the fertilization process: “a group of cells resulting from fertilization whose completion was signalled by the first cell division or the expulsion of the polar bodies” (Rivron et al, 2023, p. 3550). This type of definition is not very practical or realistic since it involves both structures that could develop into a foetus and others that could not, owing to, for example, mutations of the cellular or mitochondrial DNA. Indeed, from a legal point of view, both types of entities do not have the same importance. Nonetheless, this perspective held prominently for decades until the emergence of SCNT techniques in the 1990s and the birth of Dolly, the cloned sheep. SNCT made it possible to ‘conceive’ an adult mammal without resorting to the mixing of genetic material that occurs during fertilization.

This achievement raised two possibilities: either maintaining the traditional definition, thereby holding that Dolly never arose from an embryo, or expanding the concept of embryo so that it could not only be produced by fertilization but also through other techniques. The latter is what countries such as Australia or Germany did. Others, such as The Netherlands or Belgium, went a step further, completely obviating the way in which the embryo was generated to focus exclusively on its potentiality. Accordingly, in the Dutch norm, an embryo is “a cell or set of cells with the capacity to grow into a human” (Embryowet, 2001).

In our view, the second strategy of considering the potentiality or capacity for development makes more sense as it obviates the various ways in which a cell or group of cells can arise and instead focus on its own qualities. After all, in legal terms, an embryo is only relevant for its capacity to give rise to a person, that is, to a human being. For the law, the only relevant subject is a person. Any other entity—embryo, foetus or corpse—would only be relevant insofar as it relates to a person that has existed or may exist. Thus, if there is any legally significant characteristic, it must be the capacity to become into a human being after development and birth.

After all, in legal terms, an embryo is only relevant for its capacity to give rise to a person, that is, to a human being.

## What capacity?

The new definition based on potentiality was not without problems either. What kind of potentiality or capacity for development should be required in order to consider cells or groups of cells as embryos? That question prompted different solutions. In the USA, the Dickey-Wicker Amendment, which was passed by Congress in 1996 as part of an appropriations bill, stated that “the term ‘human embryo or embryos’ includes any organism, not protected as a human subject under 45 CFR 46 as of the date of the enactment of this Act, that is derived by fertilization, parthenogenesis, cloning, or any other means from one or more human gametes or human diploid cells” (Dickey-Wicker Amendment, 1996). Consequently, parthenotes have been considered as embryos under this rule in the USA (Rodriguez et al, 2011).

The EU adopted a similar definition. In 2011, in the context of Case C-34/10, Oliver Brüstle v Greenpeace e.V., the Court of Justice of the European Union (CJUE) ruled that “any human ovum after fertilisation, any non-fertilised human ovum into which the cell nucleus from a mature human cell has been transplanted, and any non-fertilised human ovum whose division and further development have been stimulated by parthenogenesis constitute a “human embryo”’ (point 39). The reasoning behind this conclusion was shown in points 35 and 36 of the ruling: point 35 stated that “any human ovum must, as soon as fertilised, be regarded as a ‘human embryo’ within the meaning and for the purposes of the application of Article 6(2)(c) of the Directive, since that fertilisation is such as to commence the process of development of a human being”. The same consideration should “also apply to a non-fertilised human ovum into which the cell nucleus from a mature human cell has been transplanted and a non-fertilised human ovum whose division and further development have been stimulated by parthenogenesis” (point 36). This position was due to a scientific conviction by the Court that “…although those organisms have not, strictly speaking, been the object of fertilisation, due to the effect of the technique used to obtain them they are, as is apparent from the written observations presented to the Court, capable of commencing the process of development of a human being just as an embryo created by fertilisation of an ovum can do so” (point 36).

At that time, the argument went that the possibility that a cell or group of cells begins development similar to that of an embryo created by fertilization was sufficient to equate either. In the case of the EU, however, the Court did not take long to change its position. In Case C-364/13, the Court stated that “in order to be classified as a ‘human embryo’, a non-fertilised human ovum must necessarily have the inherent capacity of developing into a human being” (point 28) and “consequently, where a non-fertilised human ovum does not fulfil that condition, the mere fact that that organism commences a process of development is not sufficient for it to be regarded as a ‘human embryo’, within the meaning and for the purposes of the application of Directive 98/44” (point 29).

## Extending the criterion set by the CJEU to fertilized eggs

One may think that the CJEU took a sensible step in modifying its own position by requiring that only entities that “have the inherent capacity of developing into a human being” should be considered human embryos. However, there was one step that the court did not take: why should we limit the requirement of possessing that inherent capacity to only non-fertilised human entities and not to fertilized ones? This only makes sense if one assumes that every fertilized egg possesses the inherent capacity to become a person, that is, a born human being. But if this is not the case, it does not seem rational to put into the same category human embryo structures that do and do not have this capacity just because some of them come from fertilization. If what matters is potentiality, the origin should be irrelevant. And if so, the definition of an embryo should be: a cell or group of cells that have the inherent capacity to develop into a human being, which is similar to the one currently in force in the Netherlands and Belgium. Nonetheless, the Netherlands is currently reforming its Embryos Act, and an important reason to revisit the definition of the human embryo was precisely that defining it in terms of potential to develop into human beings would present an epistemological challenge in relation to novel stem-cell derived entities, such as human embryo models (de Wert and Dondorp, 2022).

If what matters is potentiality, the origin should be irrelevant.

While this sounds precisely what Rivron and colleagues propose, it is not the case because of one crucial detail. Not because they introduce the additional requirement that the group of human cells must be “supported by elements fulfilling extraembryonic and uterine functions”, but because they state that the potential must be sufficient “to form a fetus” (Rivron et al, 2023). From a legal point of view, however, the foetus is a category that has little importance by itself. The fact that a group of cells can develop up to twenty weeks into pregnancy does not endow it with particular importance in regard to the law. What is important is that it can be born as a human being, that is, that it will eventually be capable of surviving outside the womb, if it is given the opportunity to develop, for example, by transferring it to a uterus.

Thus, potentiality must refer to the embryo’s capacity to develop into a human being. If it cannot reach that stage, it should not be considered an embryo, but an *embryoid* (Iltis et al, 2023). Of course, our argument does not imply that an entity that has the capacity to develop to the foetal stage or even a foetus incapable of reaching viability has no moral significance. Indeed, it seems perfectly reasonable that a potential for (foetal) sentience as an underlying and in itself relevant feature affords said entity at least some moral and legal status.

Similarly, one can argue that embryos that cannot develop into persons or embryoids, as previously mentioned could still have ‘symbolic’ value as early forms of human life, and their use should therefore not be allowed for commodity reasons. This would lead us to the conclusion that legislation ought to establish regulations aimed at safeguarding the well-being of such embryoids in order to prevent any harm. Nevertheless, it is important to note that this does not suggest any form of equivalence between embryoids and actual human embryos. The proposed action would necessitate the creation of a distinct legal framework for embryoids, which is currently lacking in the majority of legislative systems, although certain regulations pertaining to specific scenarios do exist (Iltis et al., 2023).

The proposed action would necessitate the creation of a distinct legal framework for embryoids, which is currently lacking in the majority of legislative systems

Last, we would like to stress that accepting such a definition would have important practical consequences. Indeed, its adoption by the CJEU allowed the creation of parthenotes for research on cell lines. Accepting that only a cell or group of cells that have the inherent capacity to develop into a human being is an embryo and the development of a different legal framework for non-embryonic entities, that is, embryoids, would pave the way for the creation of human-animal chimeras incapable of developing into a living creature. It would also help to clarify the legal status of cell lines derived from imperfect nuclear transfers, that is, generating embryoids, rather than embryos, because of their inability to develop into a living creature. And, of course, it would help to clarify the legal status of mitochondrial transplantation if the involved mtDNA is damaged. Thus, there are significant reasons, not only from a theoretical point of view but also from a pragmatic perspective, to adopt a single definition as we propose here.

## Conclusion

The conclusion is that, from a legal point of view, the most robust definition of human embryo reads as follows*: a cell or group of cells that have the inherent capacity to develop into a human being*. This way, we will be able to discriminate between entities that really deserve the protection we grant to embryos and those that do not. This, of course, will not settle all debates. Actually, the fact that an entity is not an embryo, but an embryoid, does not mean that it does not deserve any legal protection. As we have argued, there are strong reasons to support such protection.

We will also have to discuss whether it is lawful to alter a process so that the resulting entity does not have the capacity of an embryo (de Miguel Beriain, 2007); whether a chimeric embryo is really an embryo (de Miguel Beriain, 2011); or whether the result of genetic modification of mitochondrial DNA or a mitochondrial transplant is an embryo (de Miguel Beriain et al, 2016). And, of course, determining what exactly ‘inherent capacity’ is at any given time is not easy at all. To resolve this issue, we will need to understand the biological basis of ‘potential’ and at what point it exactly emerges. On this basis, a cell or group of cells would possess inherent capacity if it were capable of giving rise to a human being without the need to alter its biological programme, just by being in the right environment. Thus, a group of cells cultured in vitro but capable of growing until birth would be an embryo—it would be enough to transfer them to a uterus for them to give rise to a person, barring an accident—but a cell that would require, for example, modification of it genomic or mitochondrial DNA would not. These are still challenging questions that require further research.

Finally, in a world where biotechnology is advancing rapidly to the point that ectogestation is becoming feasible, as well as human-animal chimeric, these types of challenges will increase. Nonetheless, a clear and universally accepted definition of an embryo as we argued above, will help to clarify some fundamental legal questions and to advance legal certainty for research and reproductive biomedicine.

## Supplementary information

Peer Review File

## References

[CR1] Ball P (2023) What is an embryo? Scientists say definition needs to change. Nature 620(7976):928–929. 10.1038/d41586-023-02641-2 37596495

[CR2] Dickey-Wicker Amendment, *1996*, (1996) (testimony of US Congress). https://embryo.asu.edu/pages/dickey-wicker-amendment-1996

[CR3] de Miguel Beriain I (2007) La clonación, diez años después: luces y sombras de una aportación científica de primer orden. Cuadernos de Realidades Sociales 69:107–124

[CR4] de Miguel Beriain I (2011) Quimeras e híbridos:¿ Problema ético o problema para la ética? Dilemata 6:101–122

[CR5] de Miguel Beriain I, Atienza Macías E, Armaza Armaza EJ (2016) Algunas consideraciones sobre la transferencia mitocondrial:¿ un nuevo problema para la bioética? Acta Bioethica 22(2):203–211

[CR6] de Wert G, Dondorp W (2022). Dutch Embryo Act under revision. BioNews. https://www.progress.org.uk/dutch-embryos-act-under-revision/

[CR7] Embryowet, (2001) (testimony of Dutch Parliament). https://wetten.overheid.nl/BWBR0013797/2021-07-01

[CR8] Iltis AS, Koster G, Reeves E, Matthews KRW (2023) Ethical, legal, regulatory, and policy issues concerning embryoids: a systematic review of the literature. Stem Cell Res Therapy 14(1):209 10.1186/s13287-023-03448-8 PMC10441753 37605210

[CR9] Rivron NC, Arias AM, Pera MF, Moris N, M’hamdi HI (2023) An ethical framework for human embryology with embryo models. Cell 186(17):3548–3557 37595564 10.1016/j.cell.2023.07.028

[CR10] Rodriguez S, Campo-Engelstein L, Tingen C, Woodruff T (2011) An obscure rider obstructing science: the conflation of parthenotes with embryos in the Dickey–Wicker Amendment. Am J Bioethics 11(3):20–28 10.1080/15265161.2010.546472 21400380

[CR11] Villalba A, Rueda J, de Miguel Beriain Í (2023) Synthetic embryos: a new venue in ethical research. Reproduction 165(4):V1–V3 36821505 10.1530/REP-22-0416

