# Supplementary material for: Re-defining the human embryo: A legal perspective on the creation of embryos in research
Source: EMBO Rep. 2024 Jan 2;25(2):467–70. doi: 10.1038/s44319-023-00034-0 (PMC10897213; doi:10.1038/s44319-023-00034-0)
Supplement: Supplementary file 1 — Peer Review File [file 44319_2023_34_MOESM1_ESM.pdf]

EMBO reports

Re-defining the human embryo

Iñigo DE MIGUEL BERIAIN, Jon Rueda, and Adrián Villalba
DOI: 10.15252/embr.202358457

Corresponding author(s): Jon Rueda (ruetxe@gmail.com)

Review Timeline:

Submission Date:	8th Nov 23
Editorial Decision:	13th Nov 23
Revision Received:	14th Nov 23
Accepted:	7th Dec 23

Editor: Holger Breithaupt

Transaction Report: The manuscript was reviewed with board member advice.
